# *Helicobacter pylori* induced miR-362-5p upregulation drives gastric cancer progression and links hepatocellular carcinoma through an exosome-dependent pathway

**DOI:** 10.3389/fcimb.2025.1582131

**Published:** 2025-05-08

**Authors:** Jianhui Zhang, Shuzhen Liu, Juan Zhang, Mingzhu Feng, Shu Chen, Yinuo Zhang, Zekun Sun, Xinying Cao, Chao Gao, Xiaofei Ji, Huilin Zhao

**Affiliations:** ^1^ Basic Medical Sciences, Binzhou Medical University, Yantai, China; ^2^ The Second School of Clinical Medicine, Binzhou Medical University, Yantai, China; ^3^ Xu Rongxiang Regenerative Medicine Research Center, Binzhou Medical University, Yantai, China

**Keywords:** *Helicobacter pylori*, miR-362-5p, TLE4, gastric cancer, exosomes, hepatocellular carcinoma

## Abstract

**Introduction:**

*Helicobacter pylori (H. pylori)* infection induced miRNA dysregulation plays an important role in gastric cancer (GC) and exosomes mediate the spread of pathogenic effects.

**Methods:**

Expression of miR-362-5p and its clinical significance in GC were analyzed using data from TCGA. The effects of miR-362-5p on GC cells’ proliferation and migration were examined by using CCK-8, EdU, transwell and scratch assays. MKN45 xenograft model in nude mice was employed to evaluate impacts of miR-362-5p on GC progression in vivo. Target gene of miR-362-5p was screened by bioinformatic analysis and verified by using dual-luciferase assay. Exosomes from H. pylori-infected GES-1 cell (Hp-GES-EVs) were isolated and miR-362-5p inside the exosome was detected. The uptake of exosome by GC cells was observed through fluorescence imaging and exosome-mediated pathogenesis was explored. Furthermore, the transport of exosome-mediated miR-362-5p via blood was examined. The effect of exosome-carried miR-362-5p on hepatocellular carcinoma (HCC) progression was investigated by hepatocyte’s uptake, proliferation and migration assays.

**Results:**

miR-362-5p was significantly upregulated in GC tissues associated with *H. pylori* infection. Downregulation of miR-362-5p in GC cells inhibited proliferation and migration in vitro and suppressed tumor growth in vivo, counteracting H. pylori-induced carcinogenesis. TLE4 was confirmed as a direct target of miR-362-5p, and miR-362-5p/TLE4 axis implicated in H. pylori-driven neoplastic transformations in GC cells. Hp-GES-EVs mediated the transport of miR-362-5p, was absorbed by GC cells and detected at elevated levels in the serum of infected mice. Moreover, Hp-GES-EVs were diffused to liver and taken up by liver cells, enhancing HCC cell proliferation and migration by targeting TLE4.

**Conclusion:**

*H. pylori* infection upregulates miR-362-5p, facilitating GC progression via TLE4 targeting. Exosome-mediated transfer amplifies its effects, contributing to liver damage and potentially facilitating HCC.

## 1 Introduction

Gastric cancer (GC) ranks as the fifth most prevalent malignancy worldwide and is the third leading cause of cancer-related mortality globally (Sung et al., 2021). Despite advancements in therapeutic strategies, including surgical resection, chemotherapy, and radiotherapy, GC remains a significant clinical challenge, with fewer than 20% of patients surviving beyond five years after diagnosis (Smyth et al., 2020). The management of GC continues to face substantial obstacles.

*Helicobacter pylori* (*H. pylori*) infection, a key risk factor for GC, has garnered extensive research attention (Choi et al., 2020). *H. pylori*, a gram-negative bacterium with a high prevalence infecting nearly 50% of the global population, colonizes the gastric mucosa (Crowe, 2019). The pathogenic mechanisms by which *H. pylori* promotes GC development are multifaceted. Chronic infection triggers the release of virulence factors such as CagA and VacA, along with inflammatory mediators and reactive oxygen species, leading to abnormal hyperplasia and apoptosis of gastric epithelial cells, ultimately contributing to GC pathogenesis (Basso et al., 2008; Franco et al., 2008).

Beyond gastric pathologies, *H. pylori* infection has been linked to a range of extra-gastric conditions, including cardiovascular diseases (Sun et al., 2023), diabetes (Jeon et al., 2012), hepatic disorders (Okushin et al., 2018), idiopathic thrombocytopenic purpura (Frydman et al., 2015), and even neurological impairments (Xie et al., 2023). Although the precise mechanisms remain incompletely elucidated, eradication of *H. pylori* has been shown to improve clinical outcomes and prognoses associated with these systemic diseases (Cheng et al., 2019; Kato et al., 2019; Abdel-Razik et al., 2020; Kim et al., 2020).

MicroRNAs (miRNAs), endogenous non-coding RNAs ranging from 17 to 25 nucleotides, play critical roles in numerous biological processes. Dysregulated miRNA expression has been implicated in various human diseases (Kong et al., 2012). *H. pylori* infection has been shown to alter miRNA expression profiles, contributing to GC development (Belair et al., 2009; Xu et al., 2024). These findings provide valuable insights into the pathogenic mechanisms of *H. pylori* infection.

Exosomes, lipid bilayer vesicles measuring 30–100 nm, are released by various cell types and have emerged as critical mediators of intercellular communication (Raposo and Stoorvogel, 2013). They are enriched with mRNA and miRNAs, facilitating their transfer and participation in miRNA-based signaling pathways (Garcia-Martin et al., 2022). Recent studies reveal that exosomes selectively package specific miRNAs, which can functionally interact with recipient cells through fusion (Isaac et al., 2021). Thus, exosomes serve as carriers of genetic material, contributing to both local and systemic disease manifestations (Kalluri and LeBleu, 2020). Recent studies support the association of H. pylori infection with extragastric diseases via the exosomal pathway, such as Alzheimer’s disease, vascular endothelial cell injury, atherosclerosis, and colitis (Yang et al., 2019; Xia et al., 2020; Park and Tsunoda, 2022).

In this study, miR-362-5p was identified as significantly upregulated in GC cases associated with *H. pylori* infection. Cellular assays and murine experiments were performed to investigate the molecular mechanisms underlying miR-362-5p-mediated GC progression induced by *H. pylori*. Additionally, the pathogenic role of exosome-mediated overexpression of miR-362-5p was thoroughly examined.

## 2 Materials and methods

### 2.1 Cell lines and cell culture

Human immortalized gastric epithelial cells GES-1 were obtained from Beyotime Biotechnology (C6268, China). HEK293 cells, GC cell lines AGS and MKN45, hepatocellular carcinoma cells Hep G2 and Hep 3B were sourced from Cell Line Bank, Chinese Academy of Science. GES-1, HEK293, Hep G2 and Hep 3B cells were cultured in DMEM medium (Pricells, PM150210, China) supplemented with 10% (v/v) FBS (Gibco, A5669701, USA), while cells of AGS and MKN45 were cultured in RPMI 1640 (Pricells, PM150110, China) medium with 10% (v/v) FBS (Gibco). All cultures were incubated at 37°C in a humidified atmosphere enriched with 5% CO_2_.

### 2.2 *H. pylori* strains cultivation and cell infection

*H. pylori* strains 26695 and SS1 were used for cell infection assays and mice infections respectively. Both strains were cultivated on Kamani plates (OXOID, CM935B, UK) supplemented with 5% sterile defibrinated sheep blood under microaerobic conditions at 37°C. In cell infection assays, cells of 26695 strain were collected and suspended in sterile PBS, then, added into cell culture at a multiplicity of infection of 100:1. The co-incubation of bacteria and cells was performed under cell culture conditions.

### 2.3 Small interfering RNA, plasmid and cell transfection

The miR-362-5p mimics, inhibitors, and negative control (NC) (GenePharma, China) were transfected using Lipofectamine 3000 (Invitrogen, L3000015, USA) according to the manufacturer’s instructions. To overexpress TLE family member 4 (*TLE4*), *TLE4* cDNA fragments were cloned into the pcDNA 3.1 vector, with an empty pcDNA3.1 vector serving as a control. A fragment of the *TLE4* 3′-untranslated region (UTR) containing either wild-type (WT) or mutated (MUT) binding site for miR-362-5p was inserted into pmirGL0 vector. Transfection was carried out also utilizing Lipofectamine 3000.

### 2.4 Prediction of the miR-362-5p target genes

Genes predicted as targets by PITA, miRmap, microT, PicTar and miRWalk databases were selected for analysis. Heatmap was generated by using https://www.bioinformatics.com.cn (last accessed on 30 Aug 2024). TargetScan (https://www.targetscan.org/) was specifically employed to predict binding sites for miR-362-5p on the transcriptional corepressor gene *TLE4*.

### 2.5 Dual-luciferase reporter assay

A blend of 1 μL miR-362-5p mimics and 250 ng pmirGL0 WT or MUT TLE4 3′-UTR was transfected into cells that had reached approximately 80% confluence using Lipofectamine 3000. After a 48-hour interval post-transfection, the luciferase activity was gauged with a dual-luciferase reporter assay system (Vazyme, DL101-01, China). The firefly luciferase activity was rectified against the renilla activity, and the resultant data were further normalized in accordance with the luciferase activity from cells transfected with the miR-control elements.

### 2.6 RNA extraction and RT-qPCR

Total RNA was extracted with TRIzol reagent (Tsingke, TSP401, China). The miRNA 1st Strand cDNA Synthesis Kit (Vazyme, MR201-02, China) and SPARKscriptI1st Strand cDNA Synthesis Kit (Sparkjade, AG0302, China) were respectively used to reverse-transcribe miRNA and mRNA to cDNA. RT-qPCR analysis was carried out using SYBR Green reagent (TransGen, AQ132-21, China). GAPDH and U6 functioned as internal references for mRNA and miRNA correspondingly. The relative expression levels of mRNA and miRNA were determined by the 2^−ΔΔCT^ method. Primer sequences are in **Supplementary Table S1**.

### 2.7 Western blotting

The cells or tissues were lysed in RIPA buffer (Solarbio, R0020, China), with the addition of phenylmethylsulfonyl fluoride 1 mM to inhibit protein degradation. The supernatant was subsequently collected by centrifugation at 12,000 × g and 4°C for 20 minutes. A total of 30 μg of protein was then separated by SDS-PAGE and transferred onto a polyvinylidene fluoride membrane (Merck, IPVH00010, Germany). The membrane was incubated with 5% skim milk at room temperature for 2 hours to block non-specific sites. Then, the membranes were incubated successively with primary antibodies for 16–18 hours at 4°C and secondary antibodies for 1 hour. Bands were visualized using ECL (Novland, PWB-001S1, China) and captured via a Tanon imaging system (Tanon, 5200, China).

The antibodies against TLE4 and lymphoid enhancer binding factor 1 (LEF1) were purchased from Abclonel Technology (A23693, A23458, China). The anti-CD63 polyclonal antibody was procured from ELK Biotechnology (ES4255, China). The anti-tumor susceptibility gene 101 protein (Tsg101) was purchased from HUABIO (ET1701-59, China). The anti-GAPDH polyclonal antibody was obtained from Proteintech (60004-1-IG, China). Secondary antibodies, including goat-produced anti-rabbit and anti-mouse IgG, were also obtained from Abclonel Technology (AS014, AS003, China).

### 2.8 Establishment of mice infection model

Animal experiments received approval from the Ethical Committee on Animal Research at Binzhou Medical University (No: 2021-010) in accordance with ARRIVE guidelines (Kilkenny et al., 2010).

Six-week-old male C57BL/6 mice were procured from Pengyue Experimental Animal Breeding Co., Ltd. in Jinan City, China. Mice were maintained under controlled conditions with a stable temperature on a light/dark cycle of 12 hours while having free access to standard chow and water. They were randomly assigned into control group and *H. pylori*-infected group (n=5). In the *H. pylori*-infected group, mice were gavaged with PBS mixed with *H. pylori* strain SS1 (at a concentration of 1×10^9^ CFU, 0.3 mL per mouse) once every other day for 4 weeks. Control mice received PBS alone instead of bacterial suspension. All mice were euthanized via cervical dislocation after an additional 8 weeks on normal feeding. Giemsa staining (Solarbio, G1010, China) were performed on gastric mucosa samples to confirm colonization by *H. pylori*.

To assess the role of exosome-associated miRNAs in mediating liver disease due to *H. pylori* infection, GW4869 (Macklin, 6823-69-4, China), an inhibitor targeting exosome biogenesis or release, was utilized during mouse infection assays. C57BL/6 mice were divided into three experimental groups (n=5): control group, *H. pylori* group and *H. pylori*+GW4869 group. The control and *H. pylori*+GW4869 mice received GW4869 (2.5 μg/g in PBS) via intraperitoneal injection every other day throughout 30 days. The routine gavage method administered treatment in the *H. pylori* group once every other day throughout 30 days. The *H. pylori*+GW4869 group simultaneously received SSl inoculations through gavage starting twenty-four hours post initial GW4869 dose. Twenty-four hours following administration of *H. pylori* SSl, mice were euthanized and corresponding tissue samples subjected to histological analysis and protein level determination.

### 2.9 *In vivo* xenograft model

Six-week-old male BALB/c-nu mice were also purchased from Jinan Pengyue Experimental Animal Breeding Co., Ltd. (Jinan, China), randomly allocated into two experimental groups (n=5): control group and miR-362-5p antagomir group. MKN45 cells were injected subcutaneously into the right flank of all mice at a density of 1 × 10^7^ cells in 100 μL PBS. After ten days allowing tumor growth to volumes between 50-100mm³, treatment commenced.

The miR-362-5p antagomir group received the antagomir at a dose of 5 nmol per injection (GenePharma, China) every three days for five injections. Control mice received PBS as substitution. The treatments continued for 15 days, after which the mice were sacrificed, and the tumor tissues were removed, weighed, photographed, and subjected for histological analysis and protein level determination. Tumor volumes were measured by assessing length (l) and width (w) to calculate volume (V = 0.5 × l × w²).

### 2.10 CCK-8 assays

Cell proliferation was measured using the Cell Counting Kit-8 (CCK-8) (Elabscience, E-CK-A362, China). Cells were seeded in 96-well plates at a density of 2000 cells per well. Following incubation, 10% CCK-8 reagent diluted in cell culture medium was added to each well, and the plates were incubated for an additional 2 hours at 37°C. The optical density (OD) of the mixture was measured at a wavelength of 450 nm using a microplate reader (Molecular Devices, SpectraMax Plus384, USA).

### 2.11 5-Ethynyl-2′-deoxyuridine staining

A cell suspension containing 1×10^5^ cells was seeded on cell slides (Solarbio, YA0350, China) in 24-well plates and cultured for 24 hours. Proliferation of cells was quantified by using the BeyoClick™ EdU-555 Cell Proliferation Assay Kit (Beyotime, C0075S, China) in accordance with the manufacturer’s protocol. The stained cells were observed under a confocal laser microscope (Zeiss, LSM880, Germany).

### 2.12 Scratch wound assays

Wound-healing assays were conducted to analyze cells migration. Test cells were seeded in a six-well plate and cultured until reaching approximately 80% confluence. Monolayers were scratched with sterile pipette tips (10 μL), and the detached cells were rinsed off with PBS. Images of the wounds were recorded using an inverted microscope (Olympus, CKX41, Japan) at both 0 h and 24 h post-cultivation. Three random locations within each well were selected to measure scratch width and average the results.

### 2.13 Transwell assays

Cell migration was evaluated using a transwell assay. Tested cells were placed in the upper chamber of a transwell insert with an 8-μm pore size (BioFit, TCS005024, China) in a serum-free medium. The lower chamber was filled with 600 μL of DMEM or RPMI 1640 medium supplemented with 20% fetal bovine serum (FBS) as a chemoattractant. After 24 hours of incubation, migrated cells adhered to the lower surface of the transwell membrane were fixed with 4% paraformaldehyde, stained with 0.1% crystal violet, and quantified under a light microscope (Olympus, CKX41, Japan).

### 2.14 Isolation and characterization of exosomes

GES-1 cells incubated with or without *H. pylori* for 12 hours were collected and washed three times with PBS (Meilunbio, MA0015, China) to remove residual bacteria. Subsequently, cells were cultured for an additional 48 hours in fresh DMEM medium supplemented with 10% exosome-depleted FBS (ABW, AB-FBS-ED0100, China). The exosomes from the supernatant were isolated using an exosome isolation and purification kit (Umibio, UR52121, China) following the manufacturer’s instructions. The distribution of exosome size was examined through NTA using a ZetaView nanoparticle tracking analyzer (Particle Metrix, PMX-x30, Germany). The shapes of exosomes were observed via transmission electron microscopy (JEOL, JEM-1400, Japan), after fixed in paraformaldehyde (4%). Presence of exosome-specific markers, CD63 and TSG101, was determined by western blotting.

### 2.15 Detection of exosomes absorbed by the recipient cell

The uptake of GES-1-derived exosomes by GC cells (AGS, MKN45) and hepatocellular carcinoma (HCC) cells (Hep G2, Hep 3B) was assessed to evaluate potential intercellular effects exerted by exosomal contents. The recipient cells with a concentration of 3 × 10^5^ cell were cultured in a Laser confocal dish (Labgic, BS-20-GJM, China). Exosomes were labelled with PKH67 (Solarbio, D0031, China) according to a previously described method (Zhang et al., 2024) and added into the cell culture for a six-hour incubation. After washed three times with PBS, the recipient cells were collected and fixed with 4% paraformaldehyde. The cytoskeleton was labelled using Actin-Tracker Red (Beyotime, C1005, China), while the nucleus was stained with DAPI. Fluorescence emitted from the labeled cells was observed under a fluorescence microscope (Thermo, M5000, USA).

### 2.16 Tracer analysis of exosomes in mice model

The exosomes labelled with PKH67 were prepared (Zhang et al., 2024) and injected via the tail vein for tracing analysis. After 24 hours, the liver tissues were excised for cryostat sections preparation (Leica CM3050S, Germany). PKH67-labeled vesicles in corresponding tissue were observed using a confocal microscope (Zeiss, LSM 880, Germany).

### 2.17 Detection of alanine aminotransferase and aspartate aminotransferase

The levels of serum ALT and AST were measured using the Glutamic-pyruvic Transaminase Assay Kit (Solarbio, BC1555, China) and the Micro Glutamic-oxaloacetic Transaminase Assay Kit (Solarbio, BC1565, China) respectively according to the product manual.

### 2.18 Histopathology

Tissues from mice were fixed in 4% paraformaldehyde immediately after euthanasia, immersed in serial alcohol dehydration solutions, and then embedded in paraffin. Subsequently, 4 μm-thick tissue sections were cut, stained with anti-Ki67 (ABclonal, A20018, China) and E-cadherin antibodies (ABclonal, A20798, China) for initial staining, and further processed using the IHC kit (ZSGB, PV-9000, ZLI-9019, China) for subsequent staining and DAB development. Imaging was performed (Thermo, M5000, USA) finally.

### 2.19 Bioinformatic analysis

The miRNA expression data were retrieved from the HMDD v4.0 (http://www.cuilab.cn/hmdd). Heatmap was plotted by https://www.bioinformatics.com.cn (last accessed on 30 Aug 2024), an online platform for data analysis and visualization. TCGA database (https://www.cancer.gov/ccg/research/genome-sequencing/tcga) were used to analysis the expression of differential gene, survival curve analysis, and ROC analysis through the Xiantao Science (https://www.xiantaozi.com/) and UALCAN (https://ualcan.path.uab.edu/index.html).

PITA (https://tools4mirs.org/software/target_prediction/pita/), miRmap (https://mirmap.ezlab.org/ ), miRDB (http://mirdb.org/), microT (http://mirtoolsgallery.tech/mirtoolsgallery/node/1084), miRWalk (http://mirwalk.umm.uni-heidelberg.de/), and PicTar (https://pictar.mdc-berlin.de/) were used to predict targets of miR-362-5p. ENCORI (https://rnasysu.com/encori/index.php) was used to analyze co-expression for miR-362-5p and TLE4.

### 2.20 Statistical analysis

The GraphPad Prism 9.0 software is used for statistical analysis. The data are reshaped as the average value ± SEM based on at least three independent experiments. Unidirectional variance analysis and Student’s T-tests are used to analyze the statistical differences between two and two groups, respectively. The *p*-value below 0.05 is considered statistically significant.

## 3 Result

### 3.1 Overexpression miR-362-5p in GC and *H. pylori* infected tissue

**Figure 1:**
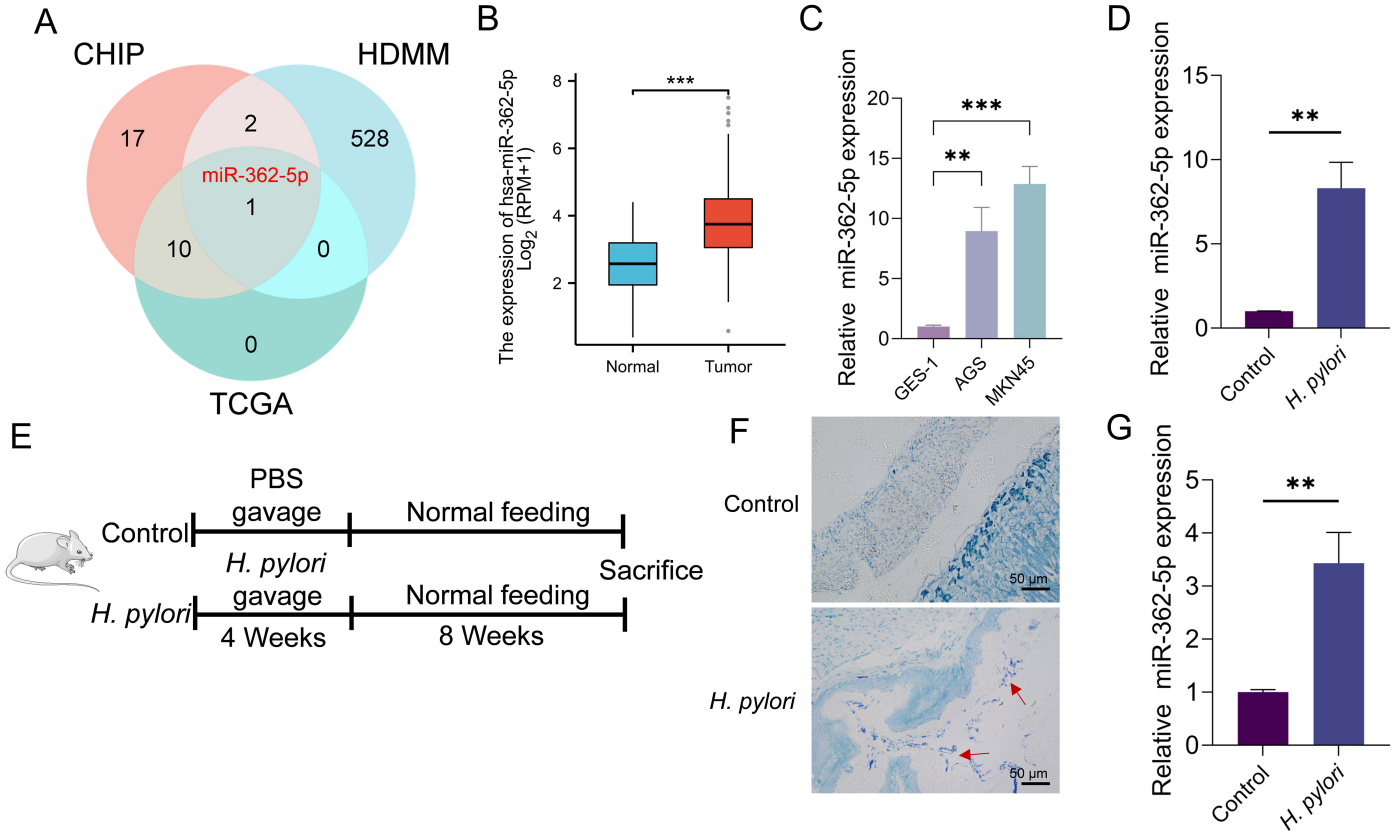
Analysis of miR-362-5p expression in GC cells and *H. pylori*-infected normal cells. **(A)** Venn diagram showing the intersection of miRNA genes identified from gene microarray analysis, the HMDD database, and the TCGA database. **(B)** Analysis of miR-362-5p expression in GC tissues and paired adjacent normal tissues by using TCGA database. **(C)** RT-qPCR detection of miR-362-5p expression in GES-1, AGS and MKN45 cells. **(D)** RT-qPCR analysis of miR-362-5p expression in GES-1 cells infected with *H. pylori*. **(E)** *H. pylori*-infected mouse model design. **(F)** Giemsa staining results of the gastric mucosa tissue of mice, and red arrows indicates *H. pylori* cells. **(G)** Expression of miR-362-5p in the gastric tissue of mice with *H. pylori* infection. Data are expressed as the mean ± SEM. **p < 0.01, ***p < 0.001. The scale bar represents 50 μm in F.

This study identified miRNAs that are differentially expressed in GC and regulated by *H. pylori* infection. Upregulated miRNAs in GC were initially retrieved from the HMDD v4.0 database (Cui et al., 2024) and intersected with miRNA microarray data derived from *H. pylori*-infected samples reported by Zhang et al. (Zhang et al., 2021). Further refinement, through cross-referencing with upregulated miRNA data for GC obtained from the TCGA database, identified miR-362-5p as the sole candidate (**Figure 1A**). Analysis of TCGA data revealed significantly elevated miR-362-5p expression in GC tissues compared to normal tissues (**Figure 1B**). This observation was further corroborated by RT-qPCR analysis, which demonstrated that miR-362-5p expression was markedly higher in GC cell lines (AGS and MKN45) compared to the normal gastric epithelial cell line GES-1 (**Figure 1C**)

The regulatory influence of *H. pylori* infection on miR-362-5p expression was further investigated. Infected GES-1 cells exhibited a positive correlation between *H. pylori* presence and miR-362-5p levels, as shown in **Figure 1D**. Additionally, a mouse infection model was established (**Figures 1E, G**), with *H. pylori* colonization confirmed via Giemsa staining (**Figure 1F**). RT-qPCR analysis of gastric mucosal tissues from the infected mice revealed increased miR-362-5p expression (**Figure 1G**). Collectively, these results indicate that miR-362-5p upregulation occurs in GC cells and in response to *H. pylori* infection, suggesting its potential involvement in the bacterium-associated pathogenesis of GC.

### 3.2 miR-362-5p promotes proliferation and migration of AGS and MKN45 cells

While previous studies have associated miR-362-5p overexpression with various malignancies, including bladder cancer (Wei et al., 2020), thyroid cancer (Li et al., 2020), hepatocellular carcinoma (HCC) (Ni et al., 2015), and acute myeloid leukemia (Ma et al., 2018), its specific role in GC progression remains unclear. To explore its functional significance, constructs for overexpression and inhibition of miR-362-5p were established in GC cell lines to evaluate its effects on cell proliferation and migration.

**Figure 2:**
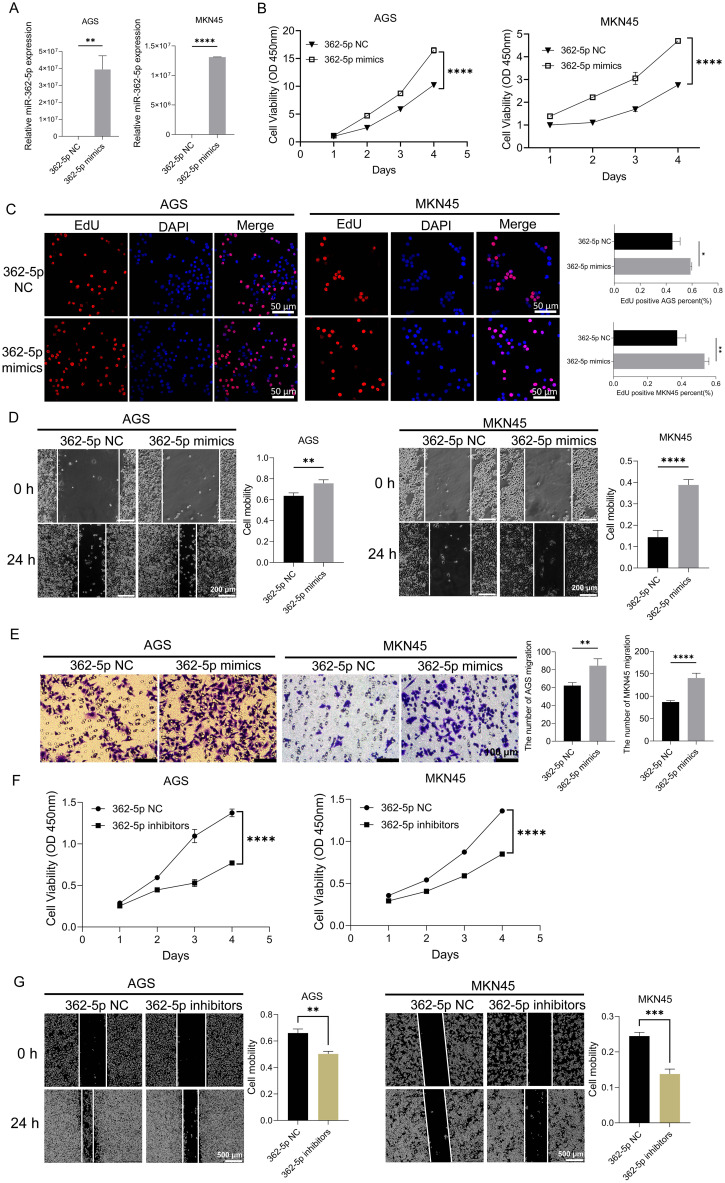
Promotion effect of miR-362-5p on proliferation and migration of GC cells. **(A)** The expression level of miR-362-5p in AGS and MKN45 cells after transfection mimics. **(B, F)** CCK-8 assays showing proliferation of AGS and MKN45 cells with the treatment of miR-362-5p mimics or inhibitors. **(C)** EdU assays showing proliferation of AGS and MKN45 cells with the treatment of miR-362-5p mimics. **(D, G)** Cell wound scratch assays assessing migration of AGS and MKN45 cells with the treatment of miR-362-5p mimics or inhibitors. **(E)** Transwell assays assessing the migration of AGS and MKN45 cells with the treatment of miR-362-5p mimics. Data are expressed as the mean ± SEM. *p < 0.05, **p < 0.01, ***p < 0.001, ****p < 0.0001. The scale bar represents 50 μm in **(C)**, 200 μm in **(D)**, 100 μm in **(E)**, and 500 μm in **(G)**.

As shown in **Figure 2A**, transfection with miR-362-5p mimics resulted in a marked increase in miR-362-5p expression in AGS and MKN45 cells. The CCK-8 assay, which evaluates cell proliferation by measuring dehydrogenase activity through absorbance at 450 nm, demonstrated significant, time-dependent increases in cell proliferation following treatment with the mimics (**Figure 2B**), indicating a promotive effect on proliferation. To further confirm these observations, EdU assays were conducted to assess the proliferative capacity of the cells. As shown in **Figure 2C**, the proportion of EdU-positive cells significantly increased after treatment with miR-362-5p mimics, consistent with the CCK-8 results. Additionally, wound healing and transwell assays were employed to evaluate the effects of miR-362-5p on cell migration. Both assays revealed a substantial increase in the number of migrated cells in AGS and MKN45 cells treated with miR-362-5p mimics (**Figures 2D, E**).

Conversely, inhibition of miR-362-5p via specific inhibitors led to a significant reduction in cell proliferation in both AGS and MKN45 cells (**Figure 2F**). Similarly, wound healing assays showed a pronounced decrease in the number of migrated cells in the inhibitor-treated groups (**Figure 2G**).

Collectively, these results demonstrate that miR-362-5p plays a critical role in promoting the proliferation and migration of GC cells.

### 3.3 miR-362-5p promotes growth and metastasis of gastric tumor *in vivo*

**Figure 3:**
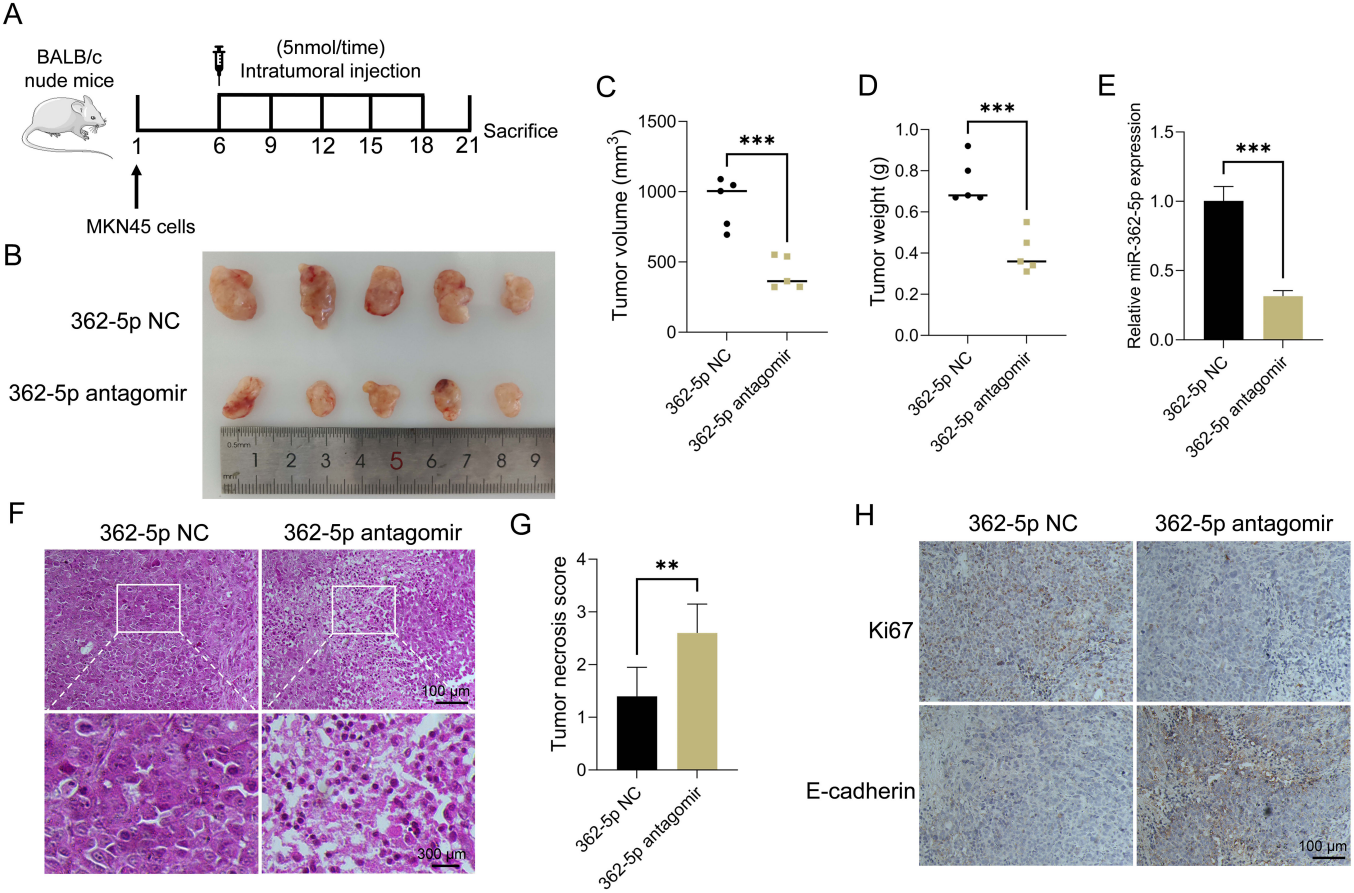
Inhibitory effect of miR-362-5p on gastric tumor growth *in vivo*. **(A)** Establishment process of mouse xenograft model using MKN45 cells. **(B)** Representative images of tumor tissues. **(C, D)** Volumes and Weights of tumors. **(E)** RT-qPCR analysis of miR-362-5p expression in tumor tissues. **(F)** HE staining of tumor sections. **(G)** Tumor necrosis scoring. **(H)** IHC analysis of Ki67 and E-cadherin expression in tumor tissues. Data are expressed as the mean ± SEM. **p < 0.01, ***p < 0.001. The scale bar represents 100 μm and 300 μm in F, and 100 μm in H.

MKN45 cells were employed to establish a mouse xenograft model. The mice were divided into two groups and received intratumoral injections of either miR-362-5p antagomir or a control solution on days 6, 9, 12, 15, and 18 following tumor formation (**Figure 3A**). On day 21 post-tumor formation, the mice were euthanized via cervical dislocation, after which the tumors were excised and photographed (**Figure 3B**). Tumor volume and weight measurements are presented in **Figures 3C, D**, respectively. Tumors in the miR-362-5p antagomir-treated group were significantly smaller than those in the control group, indicating a potential promotive role of miR-362-5p in tumor growth. RT-qPCR analysis confirmed that intratumoral injection of the miR-362-5p antagomir effectively suppressed miR-362-5p expression levels (**Figure 3E**). Histological examination with HE staining revealed dense cellular structures with minimal necrosis or apoptosis in control tumors. In contrast, tumors treated with the miR-362-5p antagomir exhibited disrupted tissue architecture, including loosely arranged cells, increased vacuolization, and substantial necrosis, indicative of enhanced cell death and cytotoxic effects (**Figure 3F**). Tumor necrosis scoring further validated the increased necrotic features observed in the miR-362-5p antagomir group (**Figure 3G**). Immunohistochemical analysis demonstrated reduced Ki67 expression, a marker of malignancy, and elevated E-cadherin levels, an epithelial marker indicative of normal tissue morphology, in the antagomir-treated group. These results suggest that miR-362-5p antagomir effectively inhibits tumor progression (**Figure 3H**).

### 3.4 *TLE4* is a direct target gene of miR-362-5p

**Figure 4:**
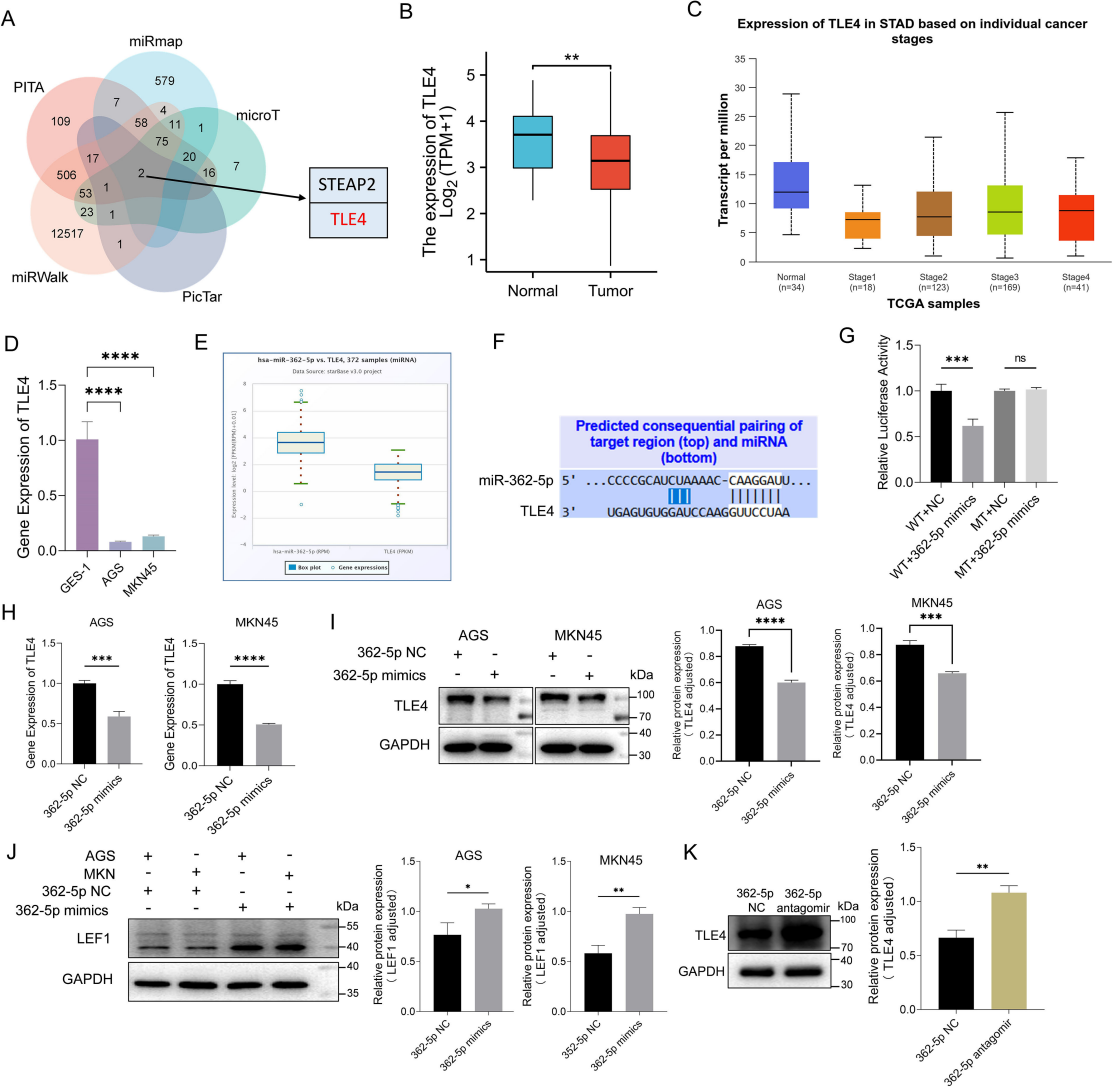
Analysis and validation of targeting genes of miR-362-5p. **(A)** Venn diagram showed the intersection of targeting genes of miR-362-5p among five datasets. **(B)** Analysis of *TLE4* expression in GC tissue using data from the TCGA database. **(C)** The expression level of *TLE4* gene across different stages (I-IV) of GC via UALCAN database. **(D)** The expression of *TLE4* in GES-1, AGS and MKN45 cell lines. **(E)** Co-expression analysis of miR-362-5p and *TLE4* by using the ENCORI database. **(F)** Illustrating of complementary sequence between miR-362-5p and the 3’-UTR of *TLE4* predicted by TargetScan. **(G)** Dual-luciferase reporter assays of HEK293 cells co-transfected with miR-362-5p mimics or miR-362-5p NC and *TLE4*-WT/MT vectors were conducted to validate the binding site. WT (wild-type), MT (mutant-type). **(H)** Transcription analysis of gene *TLE4* in addition of miR-362-5p mimics in AGS and MKN45 cells. **(I)** Expression analysis of TLE4 in addition of miR-362-5p mimics in AGS and MKN45 cells. **(J)** Expression analysis of LEF1 in AGS and MKN45 cells with miR-362-5p mimics. **(K)** Western blot analysis showing TLE4 expression in tumor tissues. Data are expressed as the mean ± SEM. *p < 0.05, **p < 0.01, ***p < 0.001, ****p < 0.0001.

To identify potential target genes of miR-362-5p, an intersection analysis was performed using five computational tools: PITA, miRmap, microT, PicTar, and miRWalk. This analysis revealed two candidate mRNAs, TLE4 and Six Transmembrane Epithelial Antigen of The Prostate 2 (STEAP2), as potential targets (**Figure 4A**). TLE4 has been implicated in the regulation of various aspects of cancer progression (Li et al., 2022) and was found to be downregulated in GC based on TCGA data (**Figure 4B**). Conversely, STEAP2 was upregulated in GC samples from the TCGA dataset, which contradicted the expected miRNA-mediated repression of mRNA (**Supplementary Figure S1A**). Thus, TLE4 was selected for further investigation.

Pan-cancer analysis using the TCGA database revealed significant downregulation of *TLE4* in multiple cancer types (**Supplementary Figure S1B**), including stomach adenocarcinoma (STAD). Further analyses using the UALCAN database (Chandrashekar et al., 2017; Chandrashekar et al., 2022) consistently showed reduced *TLE4* expression at all stages (I-IV) of GC, suggesting that low *TLE4* expression may serve as a potential predictive marker for disease progression (**Figure 4C**). Additionally, *TLE4* expression was notably lower in AGS and MKN45 cells compared to GES-1 cells (**Figure 4D**).

ENCORI database analyses revealed a negative correlation between *TLE4* and miR-362-5p expression in GC tissues, with miR-362-5p being upregulated and *TLE4* downregulated (**Figure 4E**). TargetScan predictions indicated the presence of a highly conserved binding site for miR-362-5p within the 3′-UTR of *TLE4* (**Figure 4F**).

To validate whether miR-362-5p regulates *TLE4* through this binding site, dual-luciferase reporter assays were performed. As shown in **Figure 4G**, the 3′-UTR of *TLE4* mRNA, containing the predicted miR-362-5p binding sites, was cloned into a pcDNA3.1 vector reporter plasmid (WT). Another vector containing a mutated *TLE4* 3′-UTR was also generated (MT). Co-transfection of miR-362-5p mimics with the WT luciferase vector resulted in a significant reduction in fluorescence intensity compared to the control. However, miR-362-5p mimics had no effect on cells co-transfected with the mutated *TLE4* 3′-UTR vectors. These results indicate that miR-362-5p directly targets the 3′-UTR region of *TLE4* mRNA, leading to its repression.

The regulatory effect of miR-362-5p on *TLE4* expression was further validated in GC cells. Transfection of miR-362-5p mimics led to a significant reduction in both the transcriptional and protein levels of *TLE4* (**Figures 4H, I**). Moreover, the expression of LEF1, a downstream target of *TLE4*, was inversely regulated (**Figure 4J**). In the Wnt pathway, LEF1 is a downstream protein of TLE4 and is negatively regulated by it. LEF1 has been found to be upregulated in various cancers (Santiago et al., 2017; Huang et al., 2022; Huang et al., 2023; Birdwell et al., 2024; Chen et al., 2025). Since the inhibitory effect of TLE4 on LEF1 has been previously established (Xing et al., 2018), this result further supports the role of miR-362-5p in inhibiting TLE4 expression, thereby promoting cancer progression.

Finally, the expression of TLE4 was assessed in MKN45 xenograft tumors in nude mice, revealing increased TLE4 expression in the miR-362-5p antagomir-treated group. This finding further underscores the specificity of miR-362-5p suppression of TLE4 expression (**Figure 4K**).

### 3.5 Overexpression of *TLE4* abrogates the miR-362-5p-mediated promotion of proliferation and migration in GC cells

**Figure 5:**
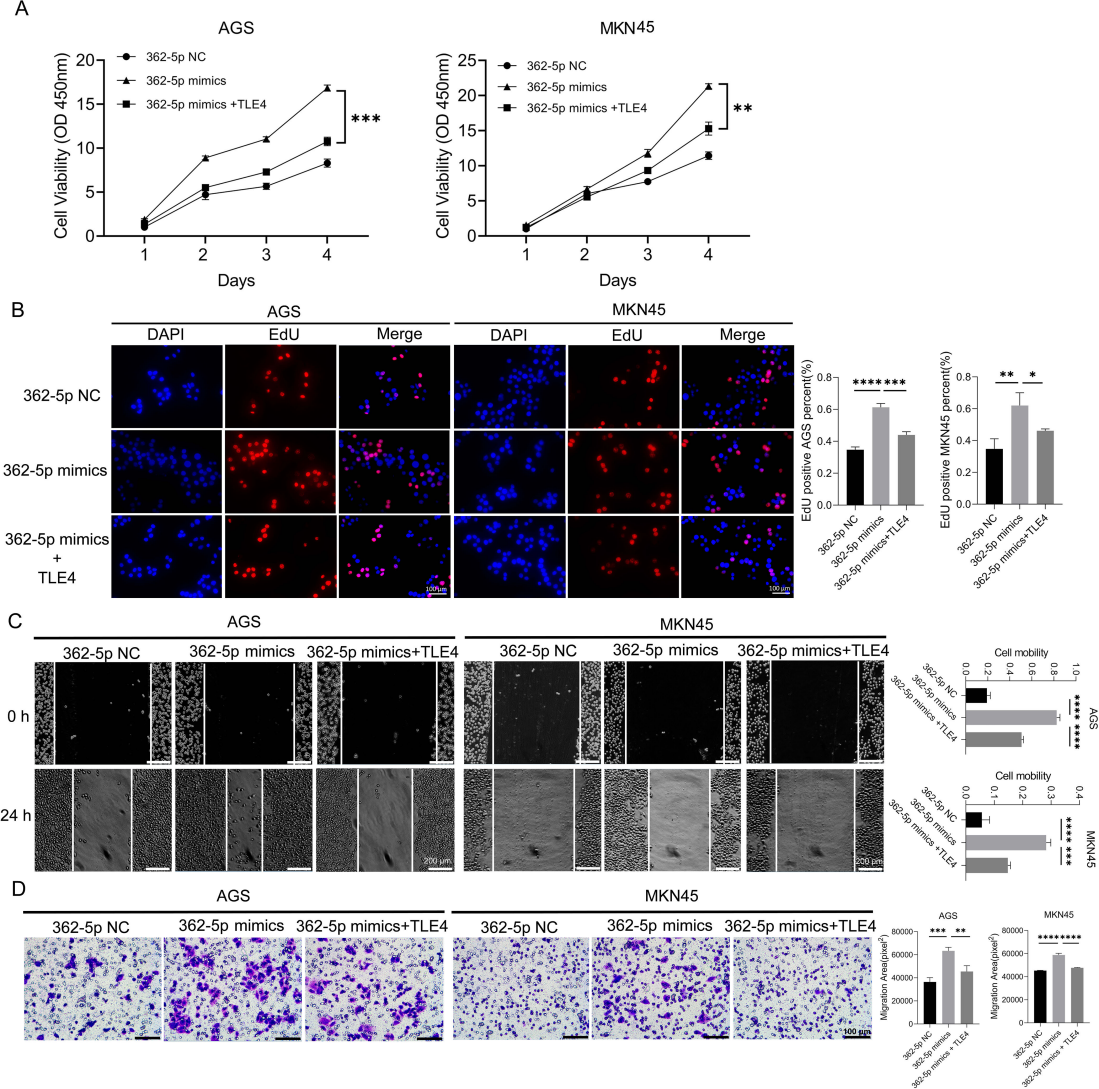
TLE4 as a target of miR-362-5p mediating the promotion of GC cell proliferation and migration. **(A)** Cell proliferation measured by CCK-8 assay in AGS and MKN45 cells. **(B)** EdU assay indicating cell proliferation. The representative images and quantitative results were shown. **(C)** Cell migration assessed by wound healing assay in AGS and MKN45 cells. **(D)** Transwell chamber were used to measure the migration ability of AGS and MKN45 cells, and representative images were shown. Data are expressed as the mean ± SEM. *p < 0.05, **p < 0.01, ***p < 0.001, ****p < 0.0001. Scale bars in **(B, D)** represent 100 μm while in **(C)** represents 200 μm.

To investigate whether TLE4 suppression mediates the enhanced malignant phenotype induced by miR-362-5p, rescue experiments were performed using a TLE4 overexpression vector devoid of miR-362-5p binding sites. Following transfection into AGS and MKN45 cells, a significant increase in TLE4 expression was observed (**Supplementary Figure S1C**). These modified cell lines were then used to assess neoplastic traits. Results from CCK-8 and EdU assays (**Figures 5A, B**) showed that, as expected, miR-362-5p mimics significantly enhanced cell proliferation. However, co-expression of the TLE4 overexpression plasmid markedly attenuated this proliferative effect, nearly abolishing the proliferation enhancement induced by miR-362-5p mimics. Wound healing assay results (**Figure 5C**) demonstrated that TLE4 overexpression significantly suppressed cell migration compared to cells treated solely with miR-362-5p mimics. Consistent findings were obtained in transwell assays (**Figure 5D**). Collectively, these data suggest that miR-362-5p promotes cell proliferation and migration through direct targeting of TLE4.

### 3.6 *H. pylori* promotes cell proliferation and migration by regulating the miR-362-5p/TLE4 axis

**Figure 6:**
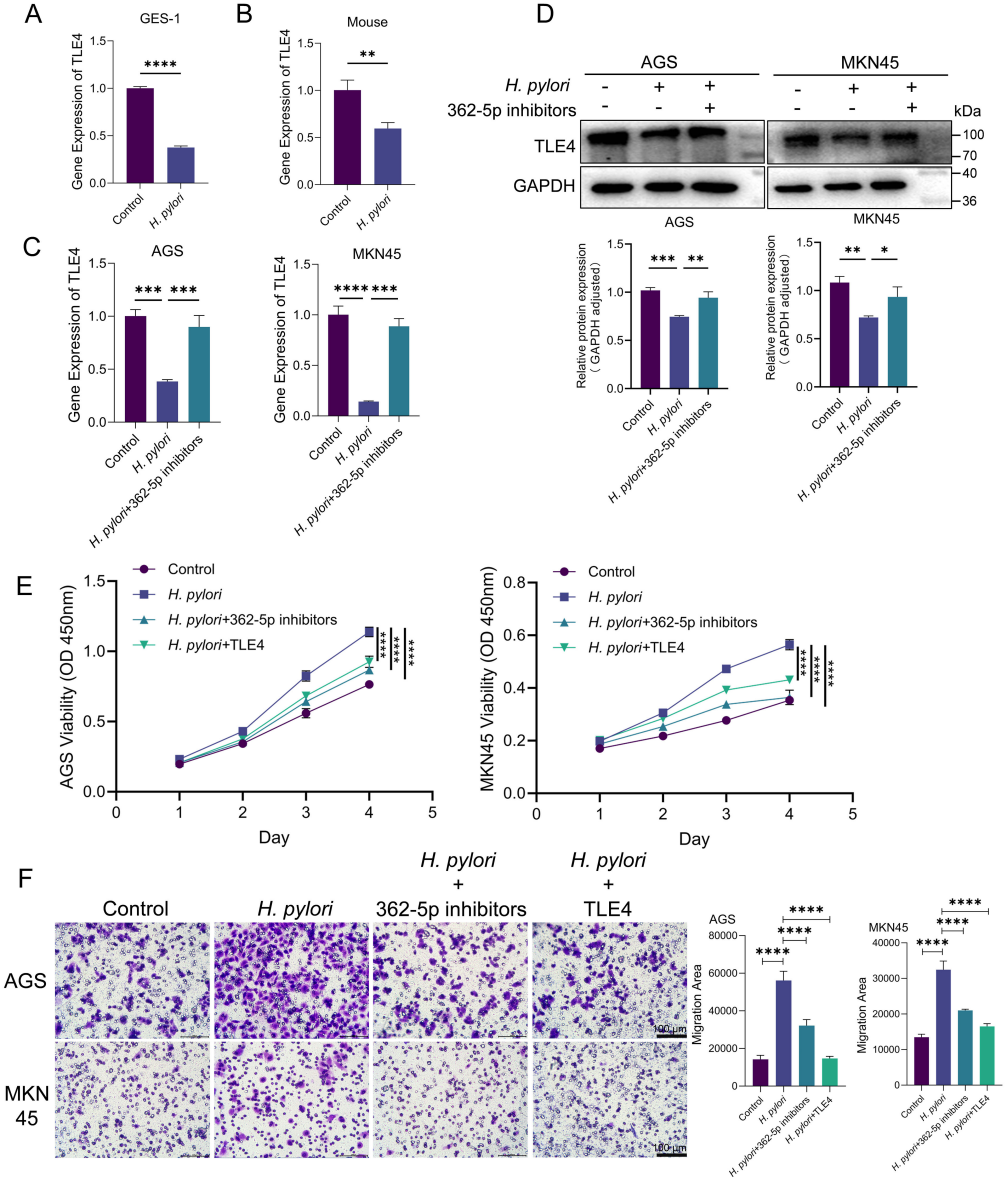
*H. pylori* promoting cell proliferation and migration via miR-362-5p/TLE4 pathway. **(A)** RT-qPCR analysis of the transcription of *TLE4* in GES-1 cells with or without *H. pylori* infection. **(B)** RT-qPCR analysis of the transcription of *TLE4* in mouse gastric tissue with *H. pylori* infection. **(C, D)** RT-qPCR and Western blot detection of TLE4 expression with the addition of miR-362-5p inhibitors in *H. pylori*-infected AGS and MKN45 cells. **(E)** Cell proliferation of AGS and MKN45 cells measured by using CCK-8 kit. **(F)** Transwell assay indicating the migration of AGS and MKN45 cells. The representative images and quantitative results were shown. Data are expressed as the mean ± SEM. *p < 0.05, **p < 0.01, ***p < 0.001, ****p < 0.0001. Scale bars in F represent 100 μm.

In *H. pylori*-infected GES-1 cells, both transcriptional activity and protein expression levels of TLE4 were found to be downregulated (**Figure 6A**). RT-qPCR analysis confirmed reduced TLE4 expression in murine models infected with *H. pylori* (**Figure 6B**). When miR-362-5p inhibitors were transfected into GC cells, TLE4 transcription and expression were partially restored (**Figures 6C, D**), suggesting that miR-362-5p negatively regulates TLE4 in response to *H. pylori* infection.

The role of the miR-362-5p/TLE4 axis in mediating the neoplastic traits induced by *H. pylori* infection was further examined. As shown in **Figure 6E**, *H. pylori* infection significantly enhanced the proliferative capacity of both AGS and MKN45 cells. Introduction of miR-362-5p inhibitors or the TLE4 overexpression plasmid resulted in reduced cell proliferation in response to *H. pylori* stimulation, indicating the involvement of the miR-362-5p/TLE4 axis in *H. pylori*-induced cell proliferation.

Transwell migration assays (**Figure 6F**) revealed that *H. pylori* infection significantly increased cell migration. However, when cells were treated with miR-362-5p inhibitors, migration was notably reduced, suggesting that miR-362-5p mediates the pro-migratory effects of *H. pylori*. Moreover, transfection of the TLE4 overexpression plasmid led to a significant reduction in *H. pylori*-induced cell migration, underscoring the inhibitory role of TLE4 on cell migration.

These results collectively establish the miR-362-5p/TLE4 axis as a critical regulatory pathway that actively contributes to the enhancement of migratory and proliferative capabilities in response to *H. pylori* infection.

### 3.7 Hp-GES-EVs regulate proliferation and migration of GC cells

**Figure 7:**
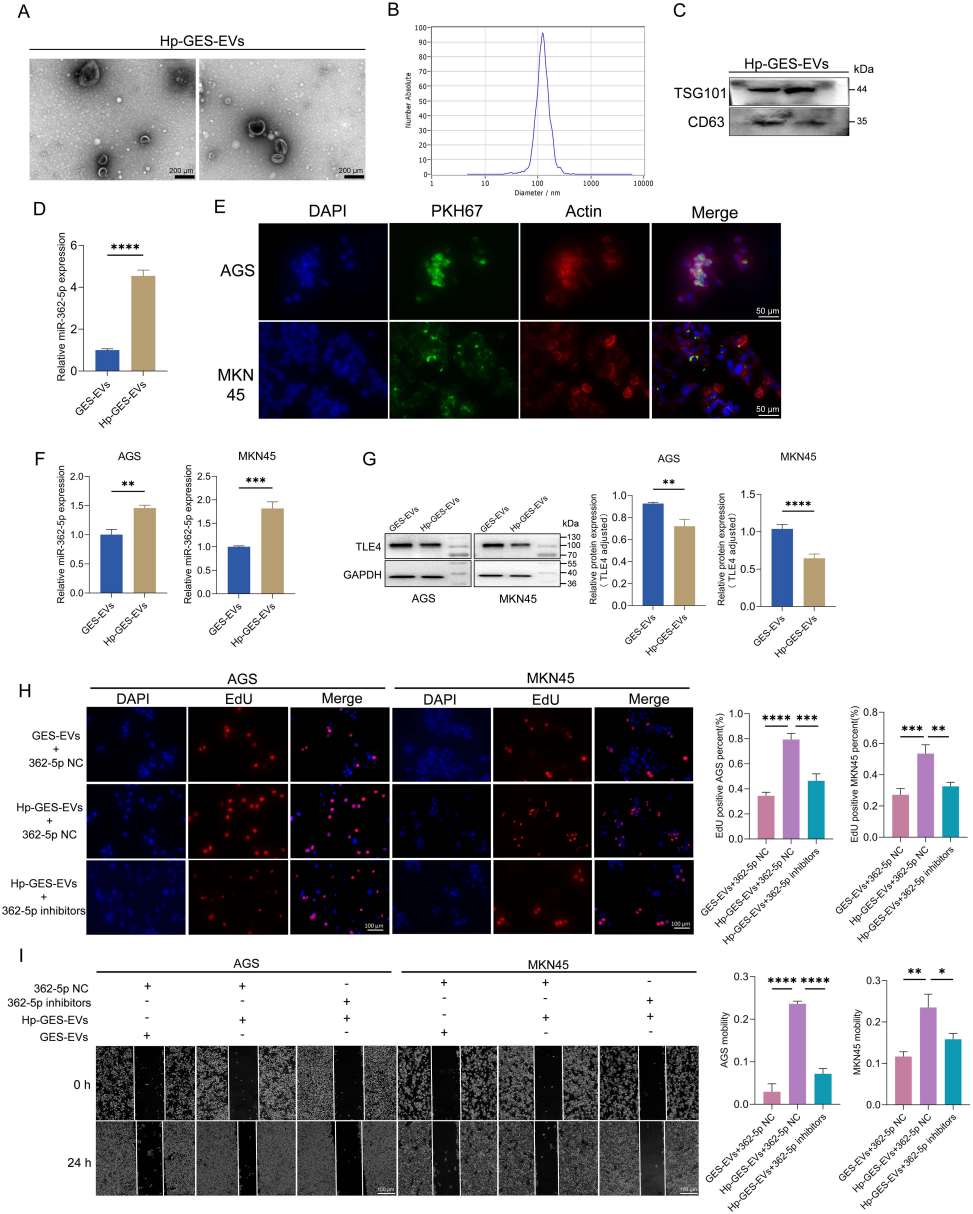
Characterization of Hp-GES-EVs and its effects on GC cells. **(A)** TEM images of exosomes isolated from Hp-GES-EVs. **(B)** NTA analysis displaying the size distribution of Hp-GES-EVs. **(C)** Western blot detection of exosomal markers TSG101 and CD63. **(D)** RT-qPCR analysis of miR-362-5p levels in Hp-GES-EVs. **(E)** Immunofluorescence analysis showing the internalization of PKH67-labeled exosomes (green) in AGS and MKN45 cells. Red fluorescence marked actin, blue fluorescence highlighted the nucleus. **(F)** RT-qPCR analysis of miR-362-5p levels in AGS and MKN45 cells after co-incubation with GES-EVs or Hp-GES-EVs. **(G)** Western blot analysis of TLE4 expression in AGS and MKN45 cells treated with GES-EVs or Hp-GES-EVs. **(H)** EdU assay showing proliferation of AGS and MKN45 cells with the action of Hp-GES-EVs and additional miR-362-5p inhibitors. **(I)** Wound healing assays showing migration of AGS and MKN45 cells with the action of Hp-GES-EVs and additional miR-362-5p inhibitors. Data are expressed as the mean ± SEM. *p < 0.05, **p < 0.01, ***p < 0.001, ****p < 0.0001. The scale bar represents 200 μm in **(A)**, 50 μm in **(E)**, 100 μm in **(H, I)**.

Research has established that exosomes can transport RNA molecules, facilitating intercellular communication and influencing tumor progression (Mashouri et al., 2019). To investigate whether the upregulation of miR-362-5p induced by *H. pylori* contributes to GC progression through an exosomal pathway, exosomes were isolated from *H. pylori*-infected GES-1 cells (termed Hp-GES-EVs). Transmission electron microscopy (TEM) revealed the characteristic cup-shaped morphology of these vesicles (**Figure 7A**), while nanoparticle tracking analysis indicated a size distribution predominantly centered around 110 nm, consistent with TEM findings (**Figure 7B**). Successful isolation was further confirmed by the presence of exosomal markers TSG101 and CD63 (**Figure 7C**).

Quantitative analysis demonstrated that Hp-GES-EVs contained significantly higher levels of miR-362-5p compared to exosomes from uninfected cells (GES-EVs) (**Figure 7D**). These miR-362-5p-enriched exosomes may represent an additional risk factor associated with *H. pylori* infection. Following a 6-hour incubation with PKH67-labeled Hp-GES-EVs (exhibiting green fluorescence), AGS and MKN45 cells displayed green fluorescence within the cytoplasm, indicating successful internalization of the exosomes (**Figure 7E**). This uptake resulted in a marked increase in intracellular miR-362-5p levels (**Figure 7F**) and a concomitant reduction in TLE4 expression, a known miR-362-5p target (**Figure 7G**). These results confirm that the miR-362-5p/TLE4 regulatory axis operates via exosome-mediated signaling during *H. pylori* infection.

The functional impact of Hp-GES-EVs on GC cells was further examined. EdU assays revealed a significant increase in the percentage of EdU-positive AGS and MKN45 cells following co-incubation with Hp-GES-EVs, indicating enhanced cell proliferation (**Figure 7H**). This proliferative effect was notably reduced upon treatment with miR-362-5p inhibitors. Similar trends were observed in wound healing assays, where Hp-GES-EVs promoted cell migration, but this effect was attenuated by the application of miR-362-5p inhibitors (**Figure 7I**).

### 3.8 miR-362-5p encapsulated within Hp-GES-EVs facilitates the progression of HCC

**Figure 8:**
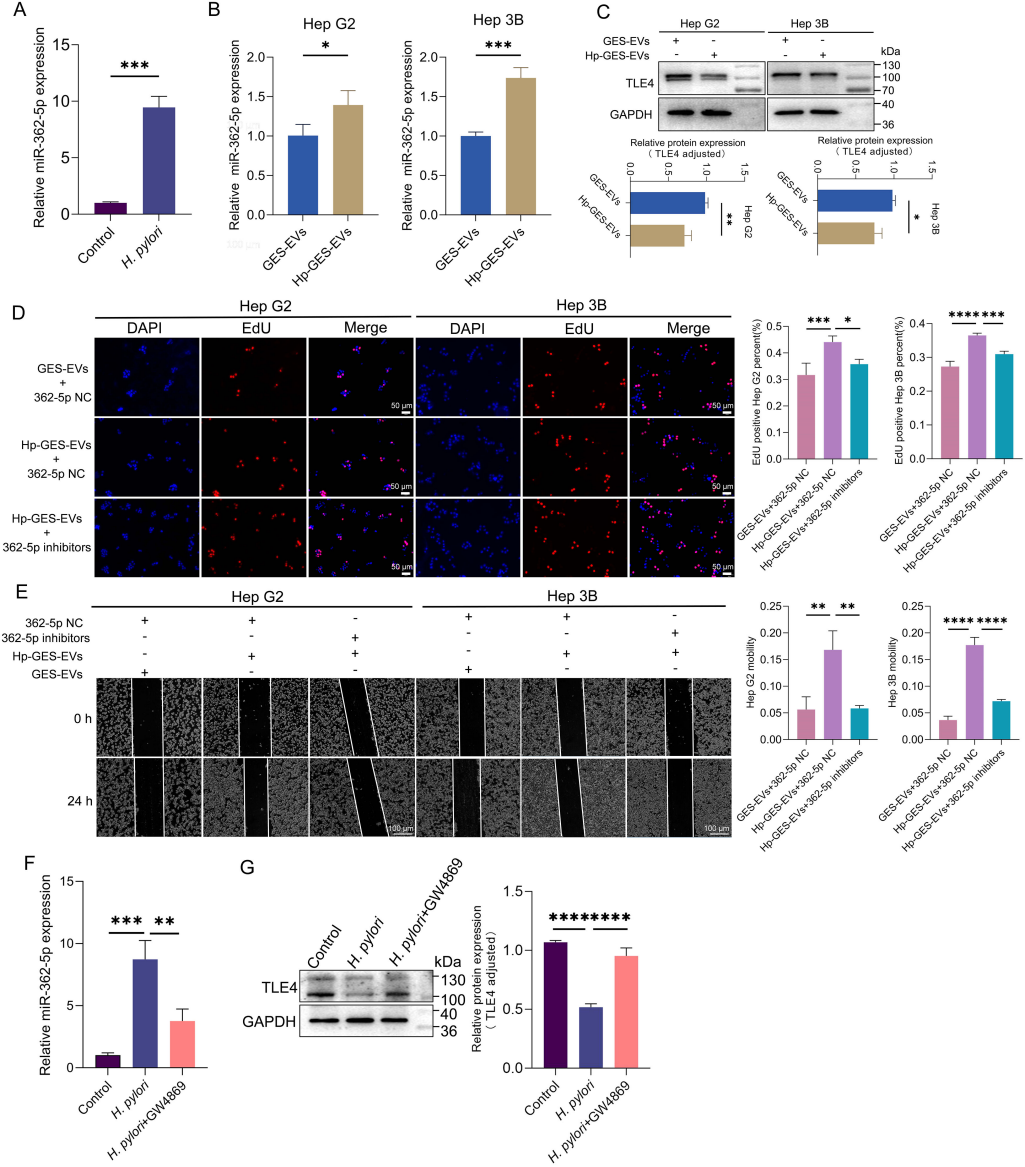
Promotion of HCC progression by Hp-GES-EVs through exosome-enclosed miR-362-5p. **(A)** miR-362-5p levels in mice serum with or without *H. pylori* infection. **(B)** RT-qPCR analysis of miR-362-5p levels in Hep G2 and Hep 3B cells co-incubated with Hp-GES-EVs. **(C)** Western blot analysis of TLE4 expression in Hep G2 and Hep 3B cells. **(D)** EdU assay showing the proliferation of Hep G2 and Hep 3B cells treated with Hp-GES-EVs and an additional miR-362-5p inhibitors. **(E)** Wound healing assay showing migration of Hep G2 and Hep 3B cells treated with Hp-GES-EVs and an additional miR-362-5p inhibitors. **(F)** serum miR-362-5p in *H. pylori*-infected mice with GW4869 treatment. **(G)** Western blot analysis of TLE4 expression in liver tissues with GW4869 treatment. Data are expressed as mean ± SEM. *p < 0.05, **p < 0.01, ***p < 0.001, ****p < 0.0001. The scale bar represents 50 μm in **(D)** and 100 μm in **(E)**.

The studies outlined above have demonstrated that exosomal miR-362-5p represents an additional risk factor in *H. pylori* infection, with the potential for systemic dissemination of its pathogenic effects via the bloodstream. Utilizing the ECONRI and GEPIA (Tang et al., 2017) database, we found that miR-362-5p was upregulated while TLE4 was downregulated in HCC tissues, suggesting the regulatory potential of miR-362-5p and TLE4 in the progression of HCC (**Supplementary Figures S2A, B**). Elevated serum levels of miR-362-5p were observed in *H. pylori*-infected mice, further supporting this hypothesis (**Figure 8A**). Previous research has shown that Hp-GES-EVs can disseminate to the liver and be absorbed by hepatocytes (Zhang et al., 2024). Consistent with these observations, co-incubation of Hp-GES-EVs with HCC cells resulted in a marked increase in miR-362-5p levels and a corresponding reduction in TLE4 expression (**Figures 8B, C**). In line with earlier observations (Zhang et al., 2024), Hp-GES-EVs enhanced cell proliferation and migration, while the application of miR-362-5p inhibitors attenuated these effects (**Figures 8D, E**).

To further investigate the pathogenic role of exosome-delivered miR-362-5p in *H. pylori* infection, a mouse model was established, and GW4869, an exosome release inhibitor, was employed. Treatment with GW4869 significantly reduced serum miR-362-5p levels, indicating that the elevated miR-362-5p primarily originates from exosomal transport (**Figure 8F**). Consistent with earlier findings (Zhang et al., 2024), *H. pylori* infection induced hepatocyte edema and degeneration, which were notably alleviated by GW4869 co-administration. Analysis of TLE4 expression in liver tissues revealed that *H. pylori* infection suppressed TLE4 expression, an effect reversed by GW4869 treatment (**Figure 8G**). These results highlight the critical role of exosome-mediated delivery of miR-362-5p in the pathogenesis of *H. pylori*-induced liver damage.

## 4 Discussion

Accumulating evidence underscores the critical roles of miRNAs in the process of carcinogenesis (Yang et al., 2018; Gen et al., 2021; Yoshida et al., 2021). *H. pylori* infection was reported to induce alterations in expression of miRNAs, which were found to be involved in carcinogenesis. This study identified miRNAs with causal roles in gastric malignancies associated with *H. pylori* infection. Bioinformatic analysis identified miR-362-5p as a candidate miRNA due to its upregulation in both *H. pylori*-infected tissues and GC specimens. Its involvement in promoting the progression of extragastric tumors has been previously reported. Ni et al. demonstrated that miR-362-5p promotes tumor growth and metastasis by targeting and inhibiting cylindromatosis (CYLD) in liver cancer cells (Ni et al., 2015). Similarly, Ma et al. highlighted the association of high miR-362-5p expression with poorer survival in cytogenetically normal acute myeloid leukemia, suggesting its utility as a novel prognostic biomarker (Ma et al., 2018). These findings underscore the pro-tumorigenic effects of miR-362-5p across various malignancies and its potential as a therapeutic target. However, its precise role in GC development remains poorly understood.

To elucidate the mechanisms underlying miR-362-5p-induced proliferation and migration of GC cells, *TLE4* was identified as a potential target gene through combined analyses. Although CYLD has also been reported as a target of miR-362-5p in liver cancer, it was not identified in our predictions. TargetScan analysis revealed that miR-362-5p shares identical binding sequences in the 3′ UTR regions of both TLE4 and CYLD. However, the luciferase reporter assay confirmed TLE4 as a direct target of miR-362-5p, a finding further validated in GC cells. Upregulation of miR-362-5p significantly reduced TLE4 expression, thereby enhancing cell proliferation and migration. Additionally, this miR-362-5p/TLE4 axis played a pivotal role in the enhanced proliferative and migratory effects observed during *H. pylori* infection. TLE4 is a member of the TLE family, a group of essential transcriptional repressors (Zhang et al., 2019). To date, seven TLE family members, designated TLE1 through TLE7, have been identified (Yu et al., 2022). These proteins interact with various transcription factors to repress target gene expression. Dysregulation of TLE proteins has been implicated in several cancer types, functioning either as oncogenes or tumor suppressors depending on the context. For instance, TLE4 is underexpressed in acute myeloid leukemia, where it acts as a tumor suppressor (Shin et al., 2016). Similarly, reduced TLE4 expression in HCC facilitates malignant proliferation and epithelial-mesenchymal transition (Wu et al., 2016). Consistent with these observations, our study demonstrates that TLE4 is downregulated in GC cells and functions as a tumor suppressor by inhibiting proliferation and migration in gastric tumors. Notably, TLE4 does not universally act as a tumor suppressor. Wang et al. reported that elevated TLE4 expression in colorectal cancer (CRC) correlates with advanced Dukes stage, lymph node metastasis, and poor prognosis (Wang et al., 2016). These findings suggest that the role of TLE4 in cancer progression is highly context-dependent, warranting further investigation to elucidate its complex involvement in oncogenesis.

The role of exosomes in inter-organ transport has been shown to be a complex and dynamic process, involving a variety of mechanisms. Existing study have demonstrated that the transport of exosomes is primarily achieved through direct fusion, endocytosis, receptor-ligand interactions, and the specific inter-organ transport characteristics via the circulatory system (Guay and Regazzi, 2017; Elliott and He, 2021; Heidarzadeh et al., 2021; Kim et al., 2024). Extensive research has demonstrated that exosomes derived from pathogen-infected cells significantly contribute to the dissemination of localized infection effects (Blaser and Berg, 2001; Schorey and Bhatnagar, 2008). Several studies have highlighted alterations in the expression of exosomal microRNAs following *H. pylori* infection, including miR-155, miR-25, and miR-124-3p. Wang et al. revealed that macrophage-derived exosomal miR-155 actively participates in inflammatory responses triggered by *H. pylori* (Wang et al., 2016). Similarly, Li et al. reported that exosomal miR-25, induced by *H. pylori*, promotes vascular endothelial cell injury (Li et al., 2019), while another study demonstrated that exosomal miR-124-3p released by *H. pylori* enhances gastric fibroblast proliferation and migration (Li et al., 2024). In the present study, it was shown that *H. pylori* infection induces elevated miR-362-5p expression, which is subsequently encapsulated within exosomes. These exosomal miR-362-5p molecules suppress TLE4 expression in recipient cells, exerting pathogenic effects through a non-bacterial contact mechanism.

The liver’s anatomical and physiological connections with other gastrointestinal organs via the hepatic portal vein create a pathway for cancer metastasis, including GC, CRC, and pancreatic cancer (Zhang et al., 2017). Growing evidence identifies *H. pylori* infection as a significant risk factor for the onset and progression of liver diseases (Huang et al., 2009; Sumida et al., 2015; Chen et al., 2024). Our previous research demonstrated that exosomes derived from *H. pylori*-infected gastric mucosal epithelial cells can infiltrate the liver and induce liver injury (Zhang et al., 2024). These exosomes enhance malignant traits in HCC cells, including increased proliferation and migration. Additional studies corroborate the role of exosomes in amplifying *H. pylori* pathogenicity beyond the gastrointestinal tract. For example, Xia et al. identified an exosome-mediated mechanism through which *H. pylori* compromises vascular endothelial integrity (Xia et al., 2020).

MiR-362-5p has been identified as an oncogenic miRNA in HCC (Ni et al., 2015; Zhao et al., 2018; Bi et al., 2025), prompting an investigation into its effects on the HCC microenvironment when derived from gastric sources. Our *in vivo* and *in vitro* results confirmed its pathogenic role in inducing liver injury. Unlike Ni et al.’s findings, which indicated that miR-362-5p promotes HCC growth and metastasis by targeting CYLD, our study pointed the regulatory role of the miR-362-5p/TLE4 axis in promoting HCC. Consistent with Wu et al.’s findings, which demonstrated that TLE4 downregulation promotes cell proliferation and epithelial-mesenchymal transition in HCC cells (Wu et al., 2016), our results suggest a connection between two critical risk factors in HCC development: miR-362-5p and TLE4. This association provides deeper insights into their roles in malignancy progression.

**Figure 9:**
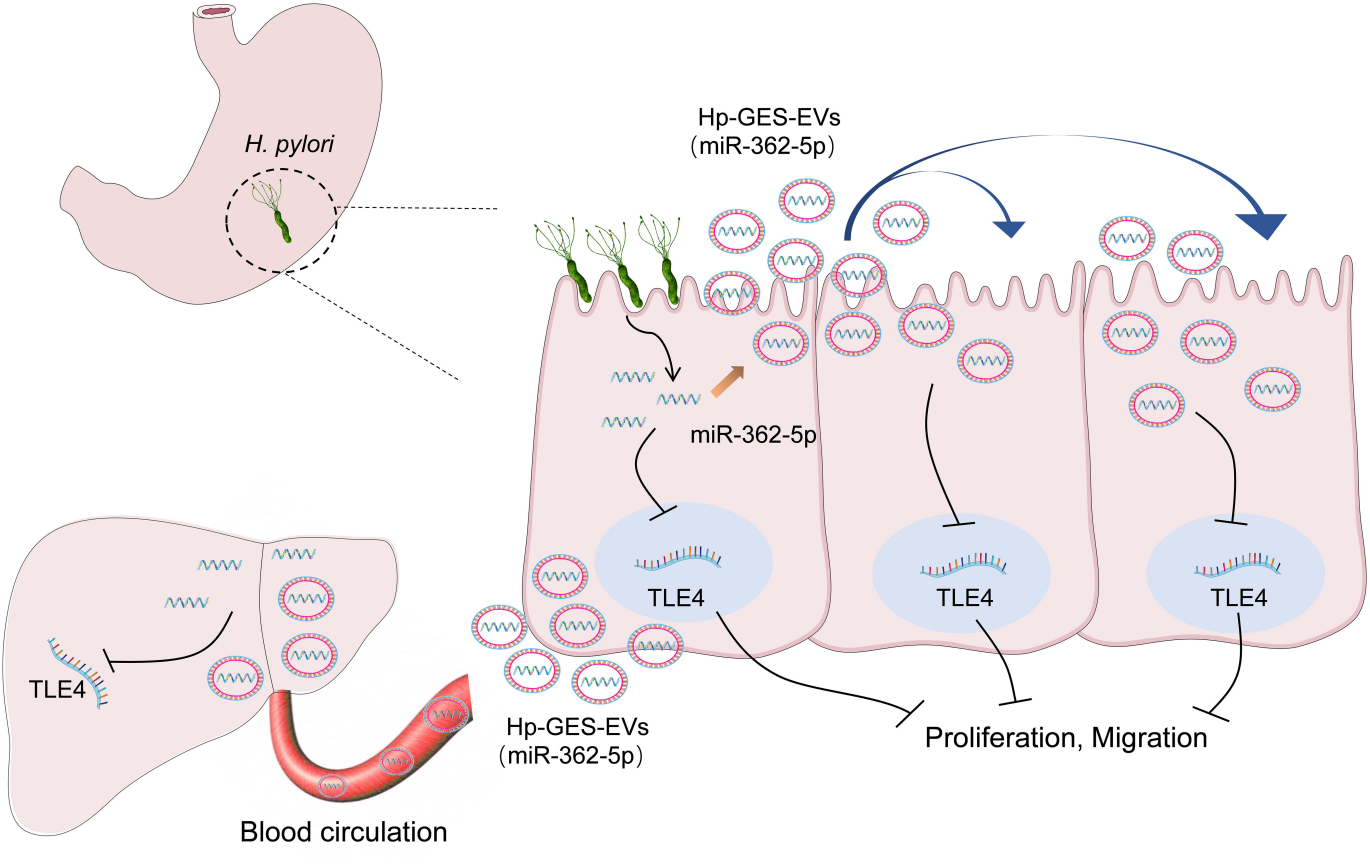
Schematic diagram of the regulatory mechanisms involved in this study.

In conclusion, this study identified miR-362-5p as a miRNA whose expression is significantly associated with *H. pylori* infection. It elucidated a novel interaction between miR-362-5p and its target gene TLE4 and demonstrated their carcinogenic role. Furthermore, the research revealed the critical role of exosome-mediated transmission in propagating the pathogenicity of miR-362-5p, offering valuable insights into the mechanisms underlying *H. pylori* infection and its related extragastric diseases (**Figure 9**).

Exosomes have a complex composition containing a variety of miRNAs as well as proteins. *H. pylori* infection causes changes in a variety of miRNAs in exosomes, and its virulence proteins such as CagA were also enveloped into the exosomes. Exosome-mediated pathogenic effects are derived from the comprehensive effects of multiple components therein. The components that function synergistically with miR-362-5p in promoting carcinogenesis need to be further explored.

## Data Availability

The original contributions presented in the study are included in the article/**Supplementary Material**. Further inquiries can be directed to the corresponding authors.
