# Supplementary material for: Helicobacter pylori induced miR-362-5p upregulation drives gastric cancer progression and links hepatocellular carcinoma through an exosome-dependent pathway
Source: Front Cell Infect Microbiol. 2025 May 8;15:1582131. doi: 10.3389/fcimb.2025.1582131 (PMC12095252; doi:10.3389/fcimb.2025.1582131)
Supplement: Supplementary file 1 [file DataSheet1.docx]

Supplementary Material

# Supplementary Figures and Tables

## Supplementary Figures

**
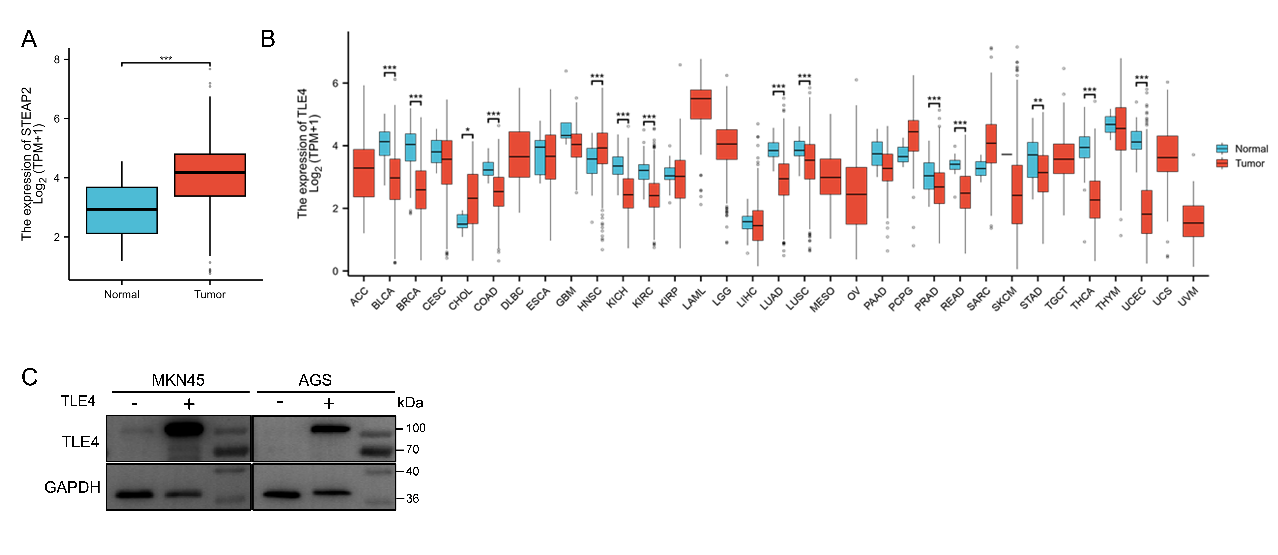
**

**Fig S1.** (A) Analysis of STEAP2 expression in GC tissue using data from the TCGA database. (B) Pan-cancer analysis of TLE4 expression (log₂(TPM+1)) in normal and tumor tissues across multiple cancer types. (C) The expression of TLE4 in MKN45 and AGS cells after transfection TLE4. Data are expressed as the mean ± SEM. **p* < 0.05, ***p* < 0.01, ****p* < 0.001.


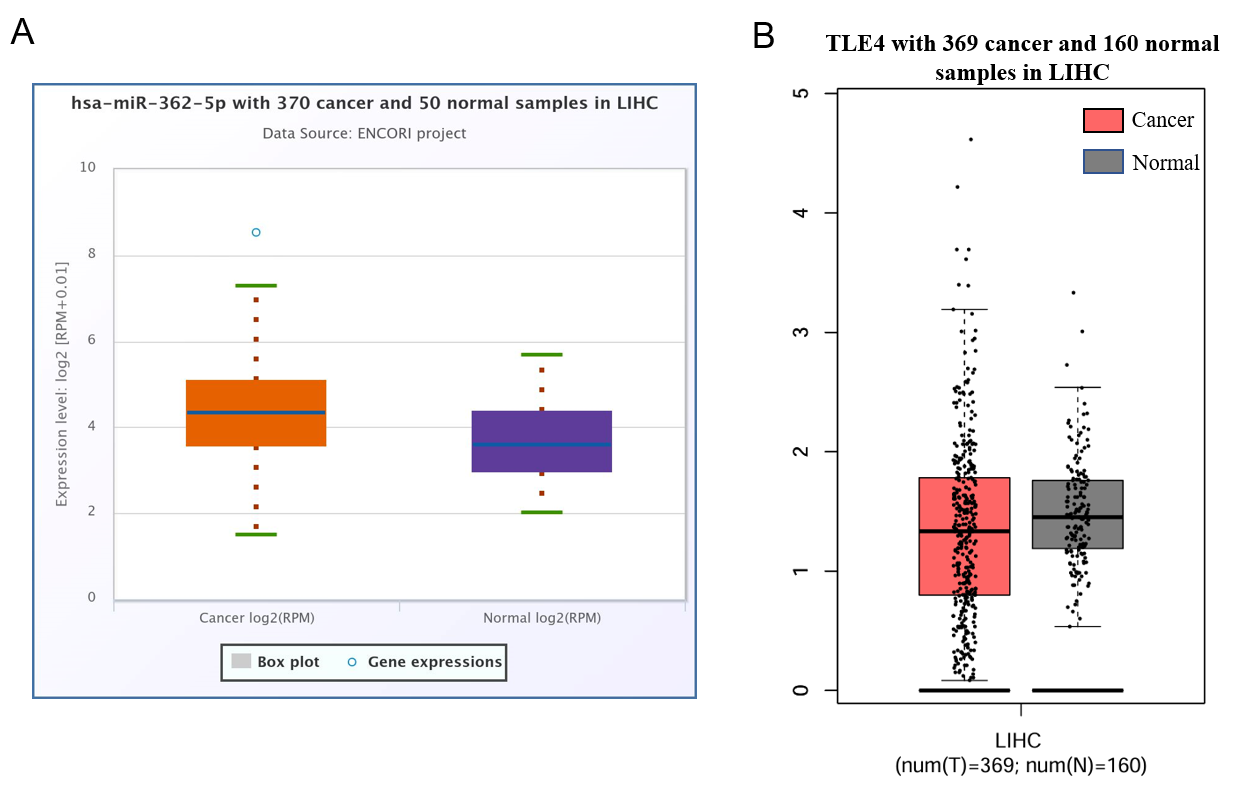


**Fig S2.** (A) Analysis of miR-362-5p expression in HCC tissues and paired adjacent normal tissues by using ENCORI database. (B) Analysis of TLE4 expression in HCC tissues and paired adjacent normal tissues by using GEPIA database.

## Supplementary Tables

**Table S1. Primer Sequences and RNA Constructs Used in the Experiment**

| Primer | Sequence |
| --- | --- |
| hsa-TLE4 | F: GTTTCCGAGGTGCTGAGAAG  R: TAATCGGGGCATCTTTCTTG |
| mmu-TLE4 | F: CCATCAGCCAGTTTCCGAGGTG  R: GCGTGTCTTGTCTAGGCCAT |
| GAPDH | F: TCCTGCACCACCAACTGCT  R: GTCAGATCCACGACGGACACA |
| U6 | F: CAGCACATATACTAAAATTGGAACG |
| mmu-miR-362-5p | F: AATCCTTGGAACCTAGGTGTGAAT |
| hsa-miR-362-5p | F: ATCCTTGGAACCTAGGTGTGAGT |
| hsa-miR-362-5p mimics | F: AAUCCUUGGAACCUAGGUGUGAGU  R: UCACACCUAGGUUCCAAGGAUUUU |
| hsa-miR-362-5p inhibitors | F: ACUCACACCUAGGUUCCAAGGAUU |
| hsa-miR-362-5p NC | F: CAGUACUUUUGUGUAGUACAA |
| hsa-miR-362-5p antagomir | F: ACUCACACCUAGGUUCCAAGGAUU |
| hsa-miR-362-5p antagomir NC | F: UUCUCCGAACGUGUCACGUTT  R: ACGUGACACGUUCGGAGAATT |
